# A comparison of medical litigation filed against obstetrics and gynecology, internal medicine, and surgery departments

**DOI:** 10.1186/s12910-015-0065-1

**Published:** 2015-10-24

**Authors:** Tomoko Hamasaki, Akihito Hagihara

**Affiliations:** Department of Nutrition Faculty of Home Economics, Kyushu Women’s University 1-1 Jiyugaoka Yahatanishi, Kitakyushu, Fukuoka, 807-8586 Japan; Department of Health Services Management and Policy, Kyushu University Graduate School of Medicine, Higashi-ku Fukuoka, 812-8582 Japan

**Keywords:** Medical litigation, Duty to explain, Departments, Obstetrics and gynecology

## Abstract

**Background:**

The aim of this study was to review the typical factors related to physician’s liability in obstetrics and gynecology departments, as compared to those in internal medicine and surgery, regarding a breach of the duty to explain.

**Methods:**

This study involved analyzing 366 medical litigation case reports from 1990 through 2008 where the duty to explain was disputed. We examined relationships between patients, physicians, variables related to physician’s explanations, and physician’s breach of the duty to explain by comparing mean values and percentages in obstetrics and gynecology, internal medicine, and surgical departments with the *t*-test and χ^2^ test.

**Results:**

When we compared the reasons for decisions in cases where the patient won, we found that the percentage of cases in which the patient’s claim was recognized was the highest for both physician negligence, including errors of judgment and procedural mistakes, and breach of the duty to explain, in obstetrics and gynecology departments; breach of the duty to explain alone in internal medicine departments; and mistakes in medical procedures alone in surgical departments (*p* = 0.008). When comparing patients, the rate of death was significantly higher than that of other outcomes in precedents where a breach of the duty to explain was acknowledged (*p* = 0.046). The proportion of cases involving obstetrics and gynecology departments, in which care was claimed to be substandard at the time of treatment, and that were not argued as breach of a duty to explain, was significantly higher than those of other evaluated departments (*p* <0.001). However, internal medicine and surgical departments were very similar in this context. In obstetrics and gynecology departments, the proportion of cases in which it had been conceded that the duty to explain had been breached when seeking patient approval (or not) was significantly higher than in other departments (*p* = 0.002).

**Conclusion:**

It is important for physicians working in obstetrics and gynecology departments to carefully explain the risk of death associated with any planned procedure, and to obtain genuinely informed patient consent.

## Background

An increase in litigation against obstetrics and gynecology departments, as compared with other medical departments, is becoming an issue in medicine [1–4]. In Japan, both maternal and perinatal mortality rates remain low [5] and healthcare safety in obstetrics and gynecology departments is considered to be excellent from a [4] global perspective. Despite this, the percentage of medical litigation cases filed against obstetrics and gynecology departments, compared with other departments, is increasing in Japan (http://www.courts.go.jp/saikosai/iinkai/izikankei/index.html). Moreover, the shortage of obstetricians and gynecologists in Japan is becoming a serious problem, along with an increase in the number of cases in which physicians have to pay substantial compensation following a medical litigation decision. This has led to the establishment of the Japan Obstetric Compensation System for Cerebral Palsy in 2011 (http://www.sanka-hp.jcqhc.or.jp/). The purpose of this system was to “provide an early resolution to disputes and compensation for the financial burden of children with severe cerebral palsy that developed in connection with delivery and their families, and to improve the quality of obstetric care.” This highlights how medical litigation in the field of obstetrics and gynecology is a growing problem in Japan and overseas.

Inappropriate explanations by physicians can lead to medical disputes [6–11]. Factors affecting physician–patient communication differ, according to the medical department, and it has been reported that poor patient–physician communication was predictive of medical claims among internists, but not among surgeons [7]. However, there has been little study of patient-surgeon communication, as compared to that involving primary physicians. Several reports contain analyses of medical litigation filed against other medical departments, including those involving family physicians [12], surgeons [13, 14], anesthesiologists [15], and radiology departments [16]. However, no studies have compared these medical departments.

To date, we have focused on physician–patient communication as the cause of medical litigation and have elucidated physician liability and its cause by analyzing medical lawsuits and quantitatively assessing physician–patient communication [17–23]. These studies have revealed that when a breach of the duty to explain is acknowledged, such cases typically involve a single physician, a clinic, and a lack of prior explanation; thus, it is clear that physicians must acknowledge that a breach of the duty to explain can easily occur in such situations.

An insufficient explanation by a physician is a breach of legal duty, even if the physician exercises sound judgment and performs a procedure perfectly. But it must be asked: What is to be explained? Earlier, the test for this was how something would have been explained by a reasonable physician in the same situation as the physician being sued [24]. However, recently, the concept of a ‘model patient’ (who is of average intelligence and not poorly educated) has gained favor. Thus, even if the physician thinks that a particular patient will not understand the explanation, the physician must carefully explain anyway [24]. Also, the field of medical ethics, and its relationship with the law, are in play. The law does not contain all of the relevant rules; the medical ethics associated with applications of medical advances, and the association between medical ethics and law, are not wholly clear. Thus, the legal extent of the duty to explain remains uncertain.

In obstetrics and gynecology, a strong relationship between medical litigation and physician’s actions has been reported [8, 25–27]. We attempted to elucidate the typical factors related to physician’s liability by comparing medical litigation regarding disputes of a breach of the duty to explain in obstetrics and gynecology with those in internal medicine and surgery. We believe that by identifying points for improvement in the explanations of obstetricians, we can provide information useful for preventing medical litigation.

## Methods

### Data source

We analyzed the outcomes of 366 medical malpractice cases reported in the *Hanrei Jiho* and *Hanrei Taimuzu*, which are major case records that report decided litigated cases in Japan, between 1990 and 2009, focusing on cases in which the pivotal issue was the physician’s duty to explain. Of these cases, we analyzed only those involving obstetrics and gynecology (62 cases), internal medicine (77 cases), and surgical (149 cases) departments.

Under the direction of one of the authors (TH), one graduate student and two students at Kyushu Dental College carefully read the case decisions. Prior training sessions were held to educate the students on the structure of a decision form, variables related to physician explanations, and patient and physician factors. One of the authors (TH) read all the decisions, and each student carefully read approximately one-third of all decisions included in the analysis. After reading the decisions, the content of each was summarized using the study variables, and a database comprising the content of each decision (*n* = 366) was constructed. To verify the validity of data coding, kappa measures of agreement were calculated with respect to the variables related to the physician’s explanation. In cases where coding was different between the raters, the cases were discussed on the basis of the coding criteria and a consensus was reached.

### Study variables

Table 1 shows the patient and physician characteristics related to litigation. Of the patient characteristics, type of treatment comprised two subcategories: elective or not urgently necessary and others, because the physician’s duty to provide an explanation to the patient was judged more severely in the field of cosmetic surgery, as compared to other fields of medicine.

**Table 1:** Comparison of study variables by department

Items	Department in which patients were treated			
	Obstetrics and gynecology	Internal medicine	Surgery	*p*-value *
*Patient characteristics*				
Age (years ± SD)	16.3 ± 19.4	37.6 ± 27.0	46.3 ± 20.7	<0.001
Gender: Male only/Male + Female or Female only	17 (27.4)/45 (72.6)	48 (62.3)/29 (37.7)	84 (57.1)/63 (42.9)	<0.001
Type of treatment: Elective or not urgently necessary/Other^a^	5 (8.1)/57 (91.9)	3 (3.9)/74 (96.1)	2 (1.3)/147 (98.7)	0.051
Severity of injury: Death/Other^b^	20 (32.3)/42 (67.7)	41 (53.2)/36 (46.8)	77 (51.7)/72 (48.3)	0.020
*Physician characteristics*				
Type of medical facility: Clinic/Hospital	21 (33.9)/41 (66.1)	14 (18.2)/63 (81.8)	12 (8.1)/137 (91.9)	<0.001
Gender: Male only/Male + Female or Female only	45 (84.9)/8 (15.1)	57 (86.4)/9 (13.6)	127 (94.1)/8 (5.9)	0.080
Number of physicians: 1/2 or more	44 (71.0)/18 (29.0)	46 (59.7)/31 (40.3)	47 (31.8)/101 (68.2)	<0.001
Medical standard: Standard care/Not standard care	40 (64.5)/22 (35.5)	61 (80.3)/15 (19.7)	139 (93.3)/10 (6.7)	<0.001
Acknowledgement of physician fault: yes/no	28 (45.2)/34 (54.8)	29(37.7)/48(62.3)	59 (39.6)/90 (60.4)	0.650
*Litigation*				
Judgment intervals: 1979–1996/1997–2008	42 (67.7)/20 (32.3)	38 (49.4)/39 (50.6)	73 (49.0)/76 (51.0)	0.034
Use of a medical expert witness: yes/no	18 (29.0)/44 (71.0)	26 (33.8)/51 (66.2)	67 (45.0)/82 (55.0)	0.058
Type of plaintiff: patient only/ patient & family, family only	8 (12.9)/54 (87.1)	10 (13.0)/67 (87.0)	51 (34.2)/98 (65.8)	<0.001
Number of issues being litigated: 0–3/4+	28 (45.2)/34 (54.8)	51(66.2)/26(33.8)	76 (51.0)/73 (49.0)	0.028
Concession rate	21.30 ± 27.29	18.15 ± 25.48	28.60 ± 52.02	0.179

* *t*-test or χ^2^ test; results printed in bold are significant (*p* <0.05)

^a^“Other” includes “treatment is urgently necessary”

^b^“Other” includes temporary or permanent injury

The type of medical facility was classified as clinic or hospital based on the Japanese medical law decision specifying that a medical institution having hospitalization facilities with more than 20 beds is defined as a hospital, and a medical institution having hospitalization facilities with 19 beds or fewer is defined as a clinic (Medical Law, Article 5, Law No. 205, 1948). Medical standard was a court judgment that determined whether a treatment was established as a medical standard. In analysis of the acknowledgement of a physician’s fault by court decision, ‘yes’ was used to indicate either a mistake in a medical maneuver or error in a physician’s judgment, or both, while ‘no’ was used to indicate other cases.

Lastly, we refer to variables related to litigation. In medical malpractice litigation, an issue related to highly professional knowledge is often included, and a medical expert witness is introduced. The introduction of a medical expert witness might affect the progress and results of litigation.

Table 4 lists variables related to a physician’s explanatory behavior. Purpose of explanation has two categories, explanation to obtain patient’s consent and others. Of these, explanation to obtain patient’s consent was related to the patient’s right of self-determination, indicating that explanation for this purpose differs from that for other purposes. Timing of physician’s explanation was divided into two categories according to whether the explanation was given prior to treatment or surgery or after treatment or surgery.

In Japanese medical settings, the family tends to play an important role when receiving the physician’s explanation. Thus, those who received the physician’s explanation comprised three categories: patient only, patient and family, and family only. Manner of physician’s explanation to patient and manner of physician’s explanation to family were both subdivided into two categories: oral only and oral and other methods, which included documents and pamphlets. Consent by the patient or family had two categories: with consent and without consent. Written consent by the patient or family was present when a consent document was present. Finally, the day of the physician’s explanation refers to the day on which the explanation was completed, categorized as the same day if the physician’s explanation was completed on the same day that the surgery or treatment was performed, and not the same day if the physician’s explanation was completed before the day that the surgery or treatment was performed.

## Results

Physician, patient, and judicial factors reported in precedents are shown in Table 1. For patient factors, we compared the degree of injury sustained by patients and found that the proportion of deaths was significantly lower for obstetrics and gynecology departments compared with internal medicine and surgical departments (*p* = 0.020). In terms of physician factors, the proportion of cases where the standard of care was not given at the time of treatment was significantly higher for obstetrics and gynecology departments, at 35.5 % (*p* <0.001). On comparing judicial factors, we found that the proportion of cases involving decisions up to 1996 was significantly higher for obstetrics and gynecology departments (*p* = 0.034).

Table 2 compares the reasons for decisions according to the medical department in cases in which the patient won, divided into the following three categories: mistakes in medical procedures and/or errors in judgment alone, breaches of the duty to explain alone, or both. We found no significant difference in the proportion of cases within the categories. The proportion of cases in which the plaintiff’s claim was recognized was highest for both categories in obstetrics and gynecology departments, while that of breaches of the duty to explain alone was highest in internal medicine departments, and mistakes in medical procedures alone highest in surgical departments (*p* = 0.008).

**Table 2:** Court decisions acknowledging the physician’s liability

	Obstetrics and gynecology (n = 62)	Internal medicine (n = 77)	Surgery (n =149)	*p*-value
Acknowledgement of physician’s fault only (%)	11 (31.4 %)	10 (22.2 %)	40 (44.9 %)	0.008
Acknowledgement of physician’s fault and a breach of physician’s duty to explain	15 (42.9 %)	16 (35.6 %)	15 (16.9 %)	
Acknowledgement of a breach of physician’s duty to explain only (%)	9 (25.7 %)	19 (42.2 %)	34 (38.2 %)	

Physician, patient, and judicial factors reported in precedents where a breach of the physician’s duty to explain occurred are shown in Table 3. Death was the most common outcome in precedents related to obstetrics and gynecology departments, when a breach of the duty to explain was acknowledged (*p* = 0.046). However, no significant difference was seen for internal medicine or surgical departments. In terms of physician factors, as for the number of attending physicians, the proportion of cases against internal medicine departments where a breach in the duty to explain was acknowledged, involving one physician, was significantly high (*p* = 0.001). However, in obstetrics and gynecology and surgical departments, no significant differences were seen in these figures. The proportion of cases involving obstetrics and gynecology departments in which the standard of care was not standard at the time of treatment that were not ruled as a breach of the duty to explain was significantly higher than in the other departments evaluated (*p* <0.001). However, no significant difference was seen for internal medicine or surgical departments. The proportion of cases involving mistakes in medical procedures or errors in judgment by physicians in obstetrics and gynecology and internal medicine departments where a breach of the duty to explain was acknowledged was significantly higher (*p* = 0.002, *p* = 0.016, respectively); however, no significant difference was seen for surgical departments.

**Table 3:** A comparison of patient and physician characteristics with litigation in the negligent and non-negligent groups regarding the physicians’ duty to explain

	Court decision with respect to a physicians’ duty to explain								
	Obstetrics and gynecology			Internal medicine			Surgery		
	Negligent	Non-negligent	*p*-value *	Negligent	Non-negligent	*p*-value *	Negligent	Non-negligent	*p*-value *
*Patient characteristics*									
Age (years ± SD)	21.3 ± 20.6	11.7 ± 18.6	0.082	39.5 ± 23.9	37.7 ± 30.1	0.793	47.1 ± 18.0	44.4 ± 22.7	0.531
Gender: Male only/Male + Female or Female only	5/20	11/20	0.164	25/10	19/18	0.081	27/22	39/33	0.919
Type of treatment: Elective or not urgently necessary/Other^a^	4/21	1/30	0.117	3/32	0/37	0.110	2/47	0/74	0.157
Severity of injury: Death/Other^b^	11/14	6/25	0.046	17/18	16/21	0.650	29/20	32/42	0.083
*Physician characteristics*									
Type of medical facility: Clinic/Hospital	11/14	7/24	0.088	8/27	5/32	0.235	6/43	5/69	0.233
Gender: Male only/Male + Female, Female only	17/3	23/5	0.558	29/1	23/8	0.015	40/4	66/3	0.264
Number of physicians: 1/2 or more	18/7	21/10	0.730	28/7	16/21	0.001	16/33	23/50	0.854
Medical standard: Standard care/Not standard care	23/2	11/20	<0.001	31/4	26/11	0.051	45/4	70/4	0.400
Acknowledgement of physicians’ fault: yes/no	16/9	7/24	0.002	17/18	21/16	0.016	17/32	18/55	0.230
*Litigation*									
The judgment year: 1979–1996/1997–2008	10/15	26/5	0.001	10/25	26/11	<0.001	20/29	41/33	0.113
Introduction of a medical expert witness: yes/no	12/13	4/27	0.005	13/22	10/27	0.358	25/24	30/44	0.252
Type of plaintiff: patient only/ patient & family, family only	5/20	2/29	0.132	7/28	3/34	0.132	20/29	25/49	0.428
Number of issue in litigation: 0–3/4+	16/9	11/20	0.034	30/5	19/18	0.002	33/16	32/42	0.009

* *t*-test or χ^2^ test; results printed in bold are significant (*p* < 0.05)

^a^“Other” includes “treatment is urgently necessary”

^b^“Other” includes temporary or permanent injury

We next investigated judicial factors and found that the proportion of recent cases involving obstetrics and gynecology and internal medicine departments where a breach of the duty to explain was acknowledged was significantly high (*p* = 0.001, *p* <0.001, respectively); however, no significant difference was seen for surgical departments. In terms of the presence or absence of a medical expert witness, the proportion of cases involving obstetrics and gynecology departments where a breach of the duty to explain was acknowledged and a medical expert witness was called was significantly high (*p* = 0.005); however, no connection was seen for internal medicine or surgical departments. The proportion of cases where a breach of the duty to explain was acknowledged and the total number of disputed points was ≤ 3 was significantly high among all departments (*p* = 0.034, *p* = 0.002, *p* = 0.009, respectively).

Table 4 compares physician’s explanatory behavior according to the presence or absence of a breach of the physician’s duty to explain. Only in obstetrics and gynecology departments were the proportion of cases significantly higher where the purpose of the explanation was to obtain the patient’s approval and when a breach of the duty to explain was acknowledged, and the proportion of cases was significantly lower where the purpose of an explanation was to obtain patient approval when a breach of the duty to explain was not conceded (*p* = 0.002). Moreover, among cases involving all departments and in which a breach of the duty to explain alone was acknowledged, the proportion of cases where the explanation given to the patient (*p* = 0.044, *p* <0.001, *p* <0.001, respectively) and family (*p* = 0.022, *p* <0.001, *p* <0.001, respectively) was not specific was significantly high.

**Table 4:** Comparison of physician’s explanatory behaviors between the negligent and non-negligent groups concerning physician’s duty to explain

	Court decision with respect to a physician’s duty to explain								
	Obstetrics and gynecology			Internal medicine			Surgery		
	Negligent	Non-negligent	*p*-value *	Negligent	Non-negligent	*p*-value *	Negligent	Non-negligent	*p*-value *
*Physician’s explanatory behavior*									
Purpose of explanation: Explanation to obtain patient’s consent/Others^a^	18/6	10/21	0.002	10/25	21/16	0.016	36/13	68/5	0.003
Issue with physician’s explanation: No explanation/Incorrect or insufficient explanation	8/16	17/14	0.112	8/27	9/28	0.884	6/43	4/69	0.159
Timing of physician’s explanation: Prior to treatment or surgery/others^b^	12/4	11/2	0.435	11/16	20/8	0.022	35/8	64/5	0.066
Who received physician’s explanation: P patien/Patient and family + Family only	4/12	4/10	0.825	13/14	12/16	0.694	11/32	20/49	0.695
Manner of physician ’s explanation to patient: Oral only/Oral and other methods	8/0	8/0	---	19/1	16/2	0.459	26/7	36/16	0.334
Manner of physician’s explanation to family: Oral only/Oral and other methods	12/0	9/0	---	13/1	15/1	0.724	25/8	33/16	0.412
Level of physician’s explanation to the patient: Relevant and specific/Not sufficiently relevant or specific	0/9	3/3	0.044	0/20	14/3	<0.001	0/33	32/16	<0.001
Level of physician’s explanation to family: Relevant and specific/Not sufficiently relevant or specific	0/10	3/2	0.022	0/13	8/3	<0.001	0/32	31/11	<0.001
Location of physician’s explanation: Inpatient ward/Outpatient clinic	12/12	23/8	0.064	6/29	18/19	0.005	35/14	57/16	0.403
Question from the patient: yes/no	8/17	7/23	0.472	14/21	7/30	0.049	19/30	16/58	0.039
Consent by the patient: with the patient’s consent/Without the patient’s consent	10/4	7/0	0.167	4/2	13/1	0.202	26/4	50/2	0.185
Consent by family: With a family’s consent/ Without a family’s consent	9/2	5/0	0.458	2/0	8/2	0.682	27/1	47/5	0.659
Written consent by the patient: Presence/Absence	3/8	0/2	0.577	0/3	4/3	0.167	11/8	15/6	0.370
Written consent by family: Presence/Absence	3/7	0/3	0.420	0/1	3/3	0.571	10/7	17/9	0.663
The day of physician’s explanation: not the same day/the same day	6/8	2/8	0.234	3/6	3/12	0.397	6/29	6/58	0.258

* *t*-test or χ^2^ test; results in bold are significant (*p* <0.05)

^a^“Other” includes explanation of medical treatment guidance and explanation of reasons for negative outcomes

^b^“Other” includes interval after treatment or surgery

## Discussion

In the present study, we focused on obstetrics and gynecology departments, for which medical litigation and substantial compensatory payouts are becoming particularly problematic, and we hypothesized that aspects of the physician’s duty to explain would differ by medical department. Several reports have emphasized the importance of communication in obstetrics and gynecology departments, [27, 28] as well as the problems associated with the continued availability of obstetric care in rural areas, caused by medical litigation [29]. We sought to define factors associated with the physician’s duty to explain by comparing obstetrics and gynecology departments with internal medicine and surgical departments. We made several interesting findings.

Firstly, we found a large number of cases in which a mistake by a physician in a medical procedure was recognized among cases involving obstetrics and gynecology departments where a breach of the duty to explain was acknowledged. While no significant differences in the proportion of cases involving mistakes in medical procedures on the part of the physician were seen among medical departments, cases where both a breach of the duty to explain and a mistake by a physician in a medical procedure were recognized were significantly more common among obstetrics and gynecology departments than other departments. We obtained different results for internal medicine departments and surgical departments; in cases involving internal medicine departments, the physician was often considered responsible when there was a breach of the duty to explain alone, whereas in cases involving surgical departments, the physician was often considered responsible when there was a mistake in a medical procedure alone. One reason for both breaches of the duty to explain and mistakes in medical procedures being recognized in cases involving obstetrics and gynecology departments may be that the patient refuses to accept the physician’s error, as stated previously. Moreover, the degree of accountability of obstetricians ranked somewhere between that of physicians working in internal medicine departments and those working in surgical departments. This may be due to interdepartmental differences in physician–patient communication, and while communication may be the cause of litigation against internal medicine departments, this may not be the case for surgical departments [7]. In other words, routine physician-patient communication differs between primary-care physicians who have and have not been required to defend prior malpractice claims. Such differences were not evident in surgeons who had been required to defend prior claims [7]. Physicians can improve communication by conversing longer with the patient, facilitating such conversation, and by being warm and friendly. Formal medical education is required to facilitate this [7]. Patients may consider surgeons to be technical experts, and thus accept a businesslike manner, but physicians are different [7]. In particular, an obstetrician is considered to be intermediate between a surgeon and a physician, and appropriate patient communication must be practiced.

Next, the number of patient deaths was significantly higher in obstetrics and gynecology departments that had conceded that a breach of the duty to explain had occurred. This is a recurring theme in medical litigation involving such departments. The extent of the injury sustained was irrelevant [6]. We also found no association between the extent of injury and the presence or absence of a breach of the duty to explain, although the numbers of negligent deaths were significantly higher in internal medicine and surgical departments than obstetrics and gynecology departments. Often, patients filing claims against obstetrics and gynecology departments consider that the serious injury complained of would not have occurred had they been fully informed about the possibility of such an outcome. Cases involving death include instances of postnatal anoxic encephalopathy, death from bacterial shock just after delivery, death from blood loss, death caused by ovarian hyperstimulation syndrome after infertility treatment, and death caused by uterine rupture after delivery. In most cases, the deaths were unexpected. However, it is very difficult to define the extent of accountability. Risk communication is problematic; if this is overdone the patient will become fearful and decline medical care that s/he urgently needs. Occupational ethics and the law related to physician behavior are in play. The topic requires further attention.

In addition, whether or not the care provided at the time of treatment was standard affected whether a breach of the duty to explain occurred in obstetrics and gynecology departments. This is likely due to retinopathy of prematurity cases in Japan from 40 years ago. A large number of medical litigation cases argued that physicians should have been aware of the fact that treating premature infants with high oxygen concentrations caused retinopathy of prematurity; thus, the medical standards at the time of treatment were disputed. In other words, a breach of the duty to explain was not acknowledged in the majority of lawsuits where a decision was made based on the year in which the case occurred, even if the decision was that the standard of care was provided. This is because physicians previously found it difficult to obtain information on medical standards. However, as this information became more widespread and we entered an era in which it was a requirement to be aware of medical standards, breaches of accountability were acknowledged. As a result, the lessons learned in obstetrics and gynecology departments may be applicable to other departments in the future. Physicians must therefore remain sensitive to daily developments and advancements in medical care and avoid neglecting their studies.

Furthermore, a particular feature of claims against obstetrics and gynecology departments is the assertion by patients of their rights to self-determination. In obstetrics and gynecology departments only, the proportion of cases in which the purpose of an explanation was to obtain patient approval for a procedure was significantly higher when a breach of the duty to explain was acknowledged than when it was not. For example, treatment and surgery applied without consent is often alleged in such instances. A typical case involved resection of a uterus with the approval of the husband, but not the patient herself; this was found to be illegal. Thus, in the absence of an emergency, the right of patient self-determination must be respected. Family relations often attend obstetrics and gynecology departments, and it is essential not to substitute an explanation to a family member for an explanation to the patient, who must herself consent.

The proportion of cases in which a breach of the duty to explain was acknowledged was significantly high among recent cases involving obstetrics and gynecology departments, and was similar to internal medicine departments. Furthermore, in internal medicine and obstetrics and gynecology departments, where physicians’ explanations carry great weight, the liability for a breach of the duty to explain was, in particularl, strongly emphasized. Moreover, the proportion of cases in which a breach of the duty to explain was acknowledged was higher among cases involving fewer disputed points, and was similar to that in internal medicine and surgical departments. Breaches of the duty to explain have not, generally, been the central issue at trials and have often been only an addition. However, several recent medical litigation cases have been filed due to breaches of the duty to explain alone, and the fact that breaches of the duty to explain tend to be acknowledged when there are fewer disputed points demonstrates their importance.

As described above, it is important that the communication style reflects what the patient expects of the medical specialist. Physicians must be accountable to their patients, and must therefore prioritize communication. Also, terminal disease, advanced age, emergency care, the need to die with dignity, euthanasia, and assisted suicide, all raise ethical issues, and such patients require different types of support. Characteristic features of surgeon-patient communication have not been described [7]. In the present study, we identified two important factors: patients value thorough explanations, and they insist on the right to make their own choices. However, as subspecialities increase in number, it will be necessary to carefully define the ethics of medical care by reference to physician skill and legal issues. In obstetricians, high medical standards are associated with good explanations. In the future, the question arises as to who will regulate the use of technology in saving lives, facilitating birth, and exploring aspects of heredity. Furthermore, will this be a matter for medical ethics, the law, or scientific associations to decide? Much further thought on such issues is required.

### Limitations of the study and future problems

This study did not deal with all recent court decisions concerning violations of the physician’s duty to explain during the study period in Japan. Thus, bias may have been introduced because the decisions were published in only two journals as case reports based on the topic and a new interpretation of the laws, and cannot easily be subjected to tests of external validity. Therefore, our results should be interpreted with caution. It was also difficult to acquire all precedents, and compromises (where cases were settled out of court) were not included in analysis.

Furthermore, surgery and internal medicine departments feature subspecialists, but this is not the case in obstetrics and gynecology departments. We did not group physicians by subspecialities, as this would have rendered analysis difficult; rather, we defined major classes of expertise. This may have affected our results. Despite these limitations, we believe that our data are useful; we extracted as much information as possible from the relevant case reports.

## Conclusion

In summary, the accountability of obstetricians was ranked between that of physicians working in internal medicine departments and those working in surgical departments. Also, it is important for physicians working in obstetrics and gynecology departments to carefully explain the risks of death to patients, to adhere to high medical standards, and to obtain patient consent.
